# New Insights in Gut Microbiota Establishment in Healthy Breast Fed Neonates

**DOI:** 10.1371/journal.pone.0044595

**Published:** 2012-08-30

**Authors:** Ted Jost, Christophe Lacroix, Christian P. Braegger, Christophe Chassard

**Affiliations:** 1 Laboratory of Food Biotechnology, Institute of Food, Nutrition and Health, ETH Zurich, Schmelzbergstrasse, Zurich, Switzerland; 2 Division of Gastroenterology and Nutrition, University Children's Hospital Zurich, Steinwiesenstrasse, Zurich, Switzerland; Institute for Genome Sciences, University of Maryland School of Medicine, United States of America

## Abstract

The establishment of a pioneer gut microbiota is increasingly recognized as a crucial stage in neonatal development influencing health throughout life. While current knowledge is mainly based on either culture or molecular analysis of feces, we opted for a comprehensive approach complementing culture with state-of-the-art molecular methods. The bacterial composition in feces from seven healthy vaginally-delivered, breast-fed neonates was analyzed at days 4–6, 9–14 and 25–30 postnatal, using culture, 16S rRNA gene sequencing of isolates, quantitative PCR and pyrosequencing. Anaerobes outnumbered facultative anaerobes in all seven neonates within the first days of life, owing to high levels of *Bifidobacterium* and unexpectedly also *Bacteroides*, which were inversely correlated. Four neonates harbored maternal *Bacteroides* levels, comprising typical adult species, throughout the neonatal period, while in three only subdominant levels were detected. In contrast, the major adult-type butyrate-producing anaerobic populations, *Roseburia* and *Faecalibacterium*, remained undetectable during the neonatal period. The presence of Bacteroidetes as pioneer bacteria in the majority of neonates studied demonstrates that adult-type strict anaerobes may reach adult-like population densities within the first week of life. Consequently the switch from facultative to strict anaerobes may occur earlier than previously assumed in breast-fed neonates, and the establishment of the major butyrate-producing populations may be limited by other factors than the absence of anaerobic conditions. The impact of breast milk components on the timing of establishment of anaerobic pioneer bacteria, as well as opportunistic pathogens should be further studied in regard to priming of the gut-associated immune system and consequences on later health.

## Introduction

The adult human gut is inhabited by up to 100 trillion indigenous bacteria belonging to over 1000 species, which constantly interact with themselves and their host [1]. Under healthy physiological conditions this microbiota-host symbiosis is generally mutualistic [2], providing the host with beneficial functions such as the metabolism of non-digestible compounds and supply of short chain fatty acids and vitamins, the prevention from colonization by pathogens, and the regulation of gut mucosal structure and immunity [3]. A dysbiotic microbiota however, although yet to be clearly defined, is increasingly associated with short- and long-term immunological disorders, including inflammatory bowel diseases, especially Crohn's disease and ulcerative colitis, and atopy [4]. As the neonatal gut is sterile, these beneficial functions are acquired concurrently with the initial colonization by pioneer bacteria, successive diversification and changes in population densities until a climax microbiota has established during infancy and early childhood – a critical time window of dynamic microbiota-host interaction profoundly influencing gut and systemic health throughout life [5], [6].

Although, this initial colonization stage is characterized by heterogeneous population dynamics, research using culture-based methods performed in the 20^th^ century led to the still widely accepted classical colonization dogma: facultative anaerobic bacteria, mainly *Staphylococcus*, *Streptococcus*, *Enterococcus* and Enterobacteriaceae spp., act as pioneer bacteria reaching high counts within the first days of life and thereby creating a reduced environment allowing the successive establishment of obligate anaerobes to dominant population levels. *Bifidobacterium* spp. are among the first anaerobes able to reach high levels in most neonates within the first to second week of life, followed by members of the Firmicutes. In contrast, high *Bacteroides* population levels are uncommon during the neonatal period, although the timing of first appearance remains not well-defined and subject to individual-specific variations [7]–[10].

These pioneer bacteria can originate from the vaginal and fecal microbiota through cross-contamination during birth, the mammary glands through breast-feeding, the skin, mouth and the environment. Thus, besides host genotype, physiological conditions and medical practices, microbiota development is profoundly influenced by the mode of delivery and gestational age [11]–[13], and the mode of feeding [14]. While, full-term vaginally-delivered, exclusively breast-fed neonates have been shown to acquire a relatively simple microbiota dominated by beneficial *Bifidobacterium* species within the first to second week of life, formula-fed neonates harbor a more diverse microbiota including Enterobacteriaceae, *Enterococcus* and *Bacteroides* [15]–[20]. In contrast to vaginal delivery, caesarean and/or pre-term deliveries lead to a delayed increase in population density of the major gut-associated anaerobes and lower ratios of anaerobes to facultative anaerobes seem to persist during infancy [13], [21].

Research using molecular methods has shown that regardless of the impact of the above-described factors, with increasing diversity the microbiota converges to one of three, so-called enterotypes with similar core bacterial populations and associated core metabolic functions during early life [22]. Nevertheless, vaginal delivery and exclusive breast-feeding during the first months of life have short- and long-term beneficial effects, such as protection against infectious diseases, reduced infant morbidity and mortality, and low incidence of immunological disorders [23]–[25]. Likely, the latter is related to differences in gut mucosal and immunity development due to relatively low (breast-fed), high (formula-fed) or delayed exposure to specific bacterial antigens (caesarean, pre-term), and the elicited pro- or anti-inflammatory responses [26]. Gaining further knowledge about the population dynamics of pioneer bacteria may provide the only opportunity for directing bacterial assembly if delayed, or for manipulating a dysbiotic microbiota in the long term (i.e. probiotics, fecal bacteriotherapy).

A number of previous studies investigating gut microbiota composition used either culture and isolation, or novel molecular methods. Culture is limited by difficulties in maintaining strictly anaerobic conditions and meeting the special nutrient requirements of fastidious bacteria, resulting in an estimated majority of up to 90% of bacteria that escape culture with the currently available techniques [27]. While, novel molecular methods such as high-throughput sequencing can partly overcome these limitations and have been applied successfully for studying human microbiota composition [26], [28]–[31], a PCR bias is inherent to these methods; and they are generally limited to bacterial identification at higher phylogenetic levels, apart from the fact that no information on viability can be gained. Thus, even with the advent of advanced molecular methods, culture and isolation still remains inevitable for studying phenotypic and genotypic characteristics of strains of interest and for developing novel probiotics. Thus in order to provide stronger evidence when studying complex microbiotas, we opted for combining culture-dependent and -independent methods.

The present study investigated the successional gut microbiota establishment during the neonatal stage in seven healthy, exclusively breast-fed neonates delivered vaginally at term, using a comprehensive analysis approach complementing anaerobic culture with state-of-the-art molecular methods, Sanger sequencing, quantitative PCR (qPCR) and pyrosequencing. The bacterial composition in neonatal feces was compared to the composition in corresponding maternal feces.

## Materials and Methods

### Participants

Healthy mothers and their neonates, delivered vaginally at term and who were exclusively breast-fed over the neonatal period, were included in this human study. Exclusion criteria were any variables known to affect the balance of the gut microbiota in either mother or neonate, such as pre-term and caesarean delivery, gastrointestinal and immunological disorders, as well as drug administration (e.g. antibiotics, laxatives) during (mother or neonate) and at least four month prior (mother) to the neonatal period. Mothers-to-be were recruited at the University Children's Hospital and the Hospital Zollikerberg, Zurich, Switzerland.

### Ethics statement

The study protocols were approved by the Ethics Committee of the University Children's Hospital Zurich, Zurich, Switzerland and informed written consent was obtained from all participants, i.e. mothers-to-be, on behalf of themselves and their neonates.

### Sampling

Neonatal and maternal feces were collected from seven mother-neonate pairs at three sampling points, between days 4–6, 9–14 and 25–30 postnatal. Additionally, maternal fecal samples were collected between weeks 2–8 antenatal. Due to the stringent inclusion criteria two mother-neonate pairs were excluded from the study after the first postnatal sampling point and 12 mothers-to-be after the prepartum sampling point.

Fresh neonatal feces were collected from diapers provided with a sterile gauze inlay to prevent liquid absorption and were transferred into a fecal collection container, while mothers were asked to defecate directly into containers. To maintain anaerobiosis and microbial viability, containers were provided with a gas generation system (Anaerocult A, Merck KGaA, Darmstadt, Germany) and transported at 4°C before processing samples within 4 h in an anaerobic chamber (Coy Laboratory Products Inc., Grass Lake, MI, USA) with an atmosphere of 85% N_2_, 10% CO_2_ and 5% H_2_ (PanGas AG, Dagmersellen, Switzerland). Fecal aliquots were prepared for immediate culture, while further aliquots were stored at −80°C prior to DNA extraction for qPCR and pyrosequencing.

### Culture and strain isolation

Fresh fecal aliquots of 0.5–1 g were used to prepare 10% (wet wt/vol) suspensions in pre-reduced anaerobic peptone water (Oxoid AG, Pratteln, Switzerland, supplemented with 0.5 g/L L-cysteine-HCl, Sigma-Aldrich Chemie GmbH, Buchs, Switzerland) and further serial 10-fold dilutions (vol/vol) of which 100 μL of appropriate dilutions were plated in duplicate on two non-selective and seven selective agar media. Media targeting anaerobic gut-associated bacterial populations, bacteroides mineral salts agar for *Bacteroides* spp. (using 5 g/L D-glucose as carbon source, VWR International, Dietikon, Switzerland) [32], Beerens agar for *Bifidobacterium* spp. [33], reinforced clostridial agar for members of the Clostridia [34] and Wilkins-Chalgren anaerobe agar for total anaerobes (Oxoid; supplemented with 0.5 g/L L-cysteine-HCl, Sigma-Aldrich), were incubated in an anaerobic chamber. On the other hand, media targeting facultative anaerobic populations, MacConkey agar no2 for Enterobacteriaceae/*Enterococcus* spp. (Oxoid), mannitol salt agar for *Staphylococcus* spp. (Oxoid) and nutrient agar for total facultative anaerobes (Oxoid) were incubated aerobically; except for *Lactobacillus* anaerobic de Man, Rogosa and Sharpe agar with vancomycin and bromocresol green (LAMVAB) targeting *Lactobacillus* spp. [35] and azide blood agar for gram-positive cocci/*Streptococcus* spp. (Oxoid), which were incubated in anaerobic jars. Plates were incubated for up to 14 days at 37°C and population levels were reported as log cfu/g feces.

Based on different morphologies, a set of colonies was isolated per sample and medium, streaked for purity and cultured in liquid media, Wilkins-Chalgren anaerobe broth for presumptive anaerobes (Oxoid; supplemented with 0.5 g/L L-cysteine-HCl, Sigma-Aldrich), tryptone soy broth for facultative anaerobes (Oxoid) and de Man, Rogosa and Sharpe broth for presumptive *Lactobacillus* spp. (Labo-Life Sàrl, Pully, Switzerland; supplemented with 0.5 g/L L-cysteine-HCl, Sigma-Aldrich). Purity was verified microscopically and finally viable isolates were maintained at −80°C in a final concentration of 20% (vol/vol) glycerol, while centrifuged cells were stored at −20°C until DNA extraction and subsequent Sanger sequencing.

### DNA extraction

DNA was extracted from pure culture cell pellets using a Wizard Genomic DNA purification kit (Promega AG, Dübendorf, Switzerland), and total DNA was extracted from 0.1–0.3 g of feces using a FastDNA SPIN Kit for Soil (MP Biomedicals, Illkirch, France) according to the manufacturers' instructions. DNA concentration and quality were assessed spectrophotometrically by absorbance measurements at 260 nm (NanoDrop 1000, Witec AG, Littau, Switzerland) and stored at −20°C prior to the molecular analyses.

### Sanger sequencing

PCR amplification of near full length 16S rRNA genes was performed using a 4∶1 mixture of forward primers 8f (5′-AGAGTTTGATCMTGGCTCAG-3′, universal) and 8f-bif (5′-AGGGTTCGATTCTGGCTCAG-3′, *Bifidobacterium*-specific) and a universal bacterial reverse primer 1391R (5′-GACGGGCGGTGTGTRCA-3′) (Microsynth AG, Balgach, Switzerland), as described previously [36]. Reactions of 50 μL contained 25 μL of 2 x MasterMix (Fermentas GmbH, Le Mont-sur-Lausanne, Switzerland), 0.1 mmol/L of each primer (-mixture) and 1 μL of template DNA diluted to 1 ng/μL with nanopure water. Thermocycling (Biometra TProfessional Thermocycler, Biolabo Scientifics Instruments SA, Chatel-St.-Denis, Switzerland) was performed with an initial denaturation at 94°C for 300 s, followed by 30 cycles of denaturation at 94°C for 30 s, annealing at 57°C for 60 s and elongation at 72°C for 30 s, and a final elongation at 72°C for 420 s. Specificity and amplicon size were verified by electrophoresis in 1.5% (wt/vol) agarose gels, and reactions were purified using an illustra GFX PCR DNA and Gel Band Purification Kit (GE Healthcare Europe GmbH, Glattbrugg, Switzerland) according to the manufacturer's instructions.

Cycle sequencing PCR was carried out in 20 μL reaction volumes with 5% (vol/vol) BigDye v3.1 (Applied Biosystems Europe BV, Zug, Switzerland), 4 μL 5 x sequencing buffer (Applied Biosystems), 1 µmol/L of reverse primer 1391R and 1 μL of purified PCR reaction template. Thermocycling (labcycler, SensoQuest GmbH, Göttingen, Germany) was performed with an initial denaturation at 96°C for 300 s, followed by 35 cycles of denaturation at 96°C for 10 s, annealing at 55°C for 20 s and elongation at 60°C for 240 s. Reactions were purified by dextran gel bead filtration (Sephadex, GE Healthcare) prior to loading 10 μL for capillary electrophoresis (ABI 3130xl DNA Analyzer, Applied Biosystems). Sequencing trace chromatograms were quality-trimmed and checked for miscalled bases using a chromatogram viewer (FinchTV v1.4.0, Geospizia Inc., Seattle, USA). The Basic Local Alignment Search Tool (BLAST) algorithm [37] was used to align sequences with the GenBank database [38], and phylogenetic assignments were based on the nearest neighbor (≥97% sequence similarity), excluding sequences deposited from uncultured samples.

### Quantitative PCR

Different qPCR assays were performed, using a 7500 Fast Real-Time PCR System with SYBR Green chemistry (Applied Biosystems), for the quantitation of the major gut-associated bacterial populations, *Bacteroides* spp., *Bifidobacterium* spp., Firmicutes, *Roseburia* spp./*Eubacterium rectale*, *Faecalibacterium prausnitzii*, *Lactobacillus*/*Leuconostoc*/*Pediococccus* spp., *Streptococcus* spp., *Staphylococcus* spp. and Enterobacteriaceae, as well as total bacteria. The corresponding primer sets targeted the 16S rRNA gene, except for the *Bifidobacterium* assay, in which the xylulose-5-phosphate/fructose-6-phosphate phosphoketolase gene (*xfp*) was targeted, as well as for the *Staphylococcus* and *Streptococcus* assays, in both of which the gene encoding the elongation factor Tu (*tuf*) was targeted (Table S1).

Each reaction mixture of 25 µL contained 12.5 μL 2 x SYBR Green PCR Master Mix (Applied Biosystems), 0.2 μmol/L of each specific primer (Microsynth) and 1 µl of 100-fold diluted template DNA (2 ng/µL). Cycling consisted of an initial heating step at 50°C for 120 s and a denaturation/Taq polymerase activation step at 95°C for 600 s, followed by 40 cycles of denaturation at 95°C for 15 s, and annealing/extension at 60°C for 60 s. Finally, high resolution melt curve analysis (HRM) was carried out at 95°C for 15 s and 60°C for 60 s, followed by 95°C for 15 s and 60°C for 15 s in order to control for amplification specificity. Fluorescence was detected at the end of each cycle and continuously during HRM. Type strain DNA for the generation of standard curves consisted of purified 16S rRNA gene amplicons of appropriate type strains, with the exception of plasmid pLME21 containing the 16S rRNA gene from *Bifidobacterium lactis* DSM10140T for the total bacteria assay, the *xfp* amplicon for the *Bifidobacterium* assay, and the *tuf* amplicon in both the *Staphylococcus* and *Streptococcus* assays (Table S1). Gene copy numbers of type strain DNA were deduced from spectrophotometric measurements, gene length and average DNA weight. Sample gene copy numbers per gram of wet feces were extrapolated from standard curves generated in triplicate in each run by linear regression of *C*_t_-values from serial 10-fold dilutions of appropriate type strain DNA.

### Pyrosequencing

High-throughput sequencing was performed on neonatal fecal DNA using a 454 Life Sciences system in combination with Titanium chemistry (Roche AG, Basel, Switzerland). Reactions were carried out at DNAVision SA (Charleroi, Belgium).

Partial 16S rRNA genes were amplified by PCR using a forward primer containing the Titanium A adaptor sequence (5′-CCATCTCATCCCTGCGTGTCTCCGACTCAG-3′), a 5-10 nt multiplex identifier sequence, and a template-specific primer sequence. The reverse primer contained the Titanium B adaptor sequence (5′-CCTATCCCCTGTGTGCCTTGGCAGTCTCAG-3′) and a template-specific primer sequence. The template-specific primer sequences (5′-AGGATTAGATACCCTGGTA-3′ and 5′-CRRCACGAGCTGACGAC-3′) allowed targeting the V5–V6 hypervariable 16S rRNA region [30]. Each reaction mixture of 100 µL contained 20 μL of 5x KAPA HiFi Fidelity buffer, 2U of KAPA HiFi Hotstart DNA polymerase, 0.3 mM of each dNTP (Kapa Biosystems, Woburn, MA, USA), 300 nM of each primer (Eurogentec, Liege, Belgium), and 60 ng of template DNA. Thermocycling was performed with an initial denaturation step at 95°C for 5 min, followed by 25 cycles of denaturation at 98°C for 20 s, annealing at 56°C for 40 s, and extension at 72°C for 20 s, with a final extension of 5 min at 72°C. Specificity and amplicon size were verified by electrophoresis in 1% (wt/vol) agarose gels, and amplicons were purified using a Wizard SV Gel and PCR Clean-up System (Promega) according to the manufacturer's instructions.

Amplicons were quantitated using a Quant-iT PicoGreen dsDNA assay kit (Life Technologies Corporation, Carlsbad, CA, USA) according to the manufacturer's instructions and combined in equimolar concentrations for multiplexing. The final pool of DNA was purified using an Agencourt AMPure XP system (Agencourt Bioscience Corporation, Beverly, MA, USA) according to the manufacturer's instructions and resuspended in 100 µL of Tris-EDTA buffer. Unidirectional pyrosequencing was then carried out using Primer A on a 454 Life Sciences Genome Sequencer GS FLX instrument (Roche) following Titanium chemistry.

Sequence quality was then verified according to the criteria: maximum of one mismatch in the barcode and primer, length of at least 240 nt and a maximum of two undetermined bases per sequence (excluding barcode and primers). The dataset has been deposited to the National Center for Biotechnology Information (NCBI) Sequence Read Archive (SRA) under accession number SRA050015. Phylum-, family- and genus-level taxonomic assignments of sequences that passed quality control were made using the Ribosomal Database Project (RDP) Bayesian classifier (v 2.1) [39] with a confidence threshold of 80%. The Mothur software package [40] was used for nearest neighbor clustering of the sequences into operational taxonomic units (OTU), based on which Chao1 richness and Shannon diversity estimations were calculated (Table S2).

### Statistical analysis

Quantitative data obtained from culture (duplicates) and qPCR (triplicates) were averaged and log_10_ transformed. Mean log-transformed values and relative abundance data from pyrosequencing of neonatal faeces were calculated at each time point (mean ± SD). Data obtained for maternal faeces were averaged regardless of sampling point, since no significant variations in individual bacterial populations were detected over time. Means were compared using Student's t-test and non-parametric Wilcoxon/Kruskal-Wallis tests with significance levels of *P<*0.05 using JMP statistical software (Version 9, SAS Institute Inc., Cary, NC, USA).

## Results

### Culture, isolation and quantitative PCR

**Figure 1:**
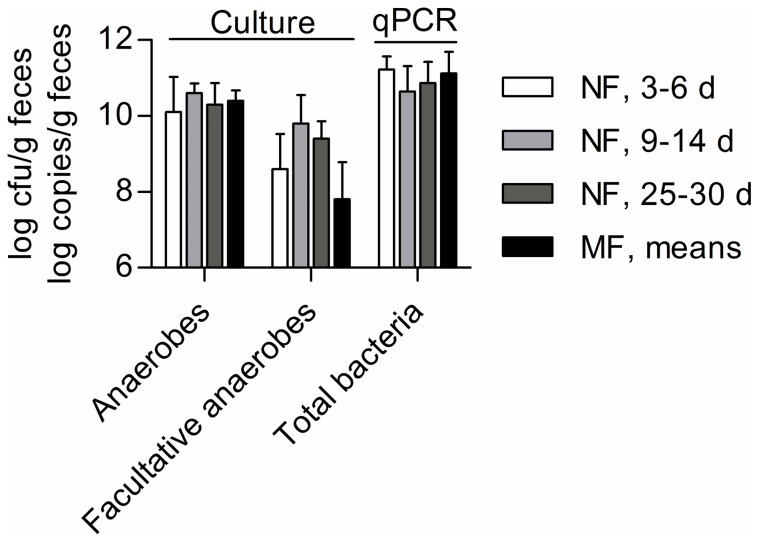
Counts of anaerobes and facultative anaerobes (culture) and total bacteria (qPCR) detected in neonatal feces (NF). Values are expressed as means ± SD at each of the three sampling points, i.e. days 4–6, 9–14 and 25–30 postnatal (*n* = 7); and compared to means obtained from maternal feces (MF) (*n* = 7) over the perinatal period.

Quantitation of the major gut-associated bacterial populations in feces by both culture and quantitative PCR revealed that a highly dense microbiota had established in all neonates by days 4–6 of life: average viable cell counts on Wilkins-Chalgren agar targeting total anaerobes and total bacteria assessed by qPCR reached 10.4±0.4 log cfu/g and 11.2±0.3 log 16S rRNA gene copies/g feces respectively (Figure 1). These population levels remained relatively stable throughout the neonatal period and did not differ significantly from maternal levels (*P = *0.94 and *P = *0.68, respectively), which were averaged for all sampling points and volunteers, since no significant variations were detected over time. Despite individual-specific and heterogeneous pattern of population densities (Figure S1), the predominance of presumptive total anaerobes over presumptive total facultative anaerobes, targeted on nutrient agar by a mean factor >60, was common to all neonates. Quantitation by qPCR showed that this predominance was largely due to high levels of *Bifidobacterium* that reached a mean of 10.36±0.77 log *xfp* copies/g feces during the first week, which was significantly higher than mean levels in maternal feces (8.87±0.58 log *xfp* copies/g, *P<*0.01). Typing of isolated strains (*n* = 197) using partial 16S rRNA gene sequencing resulted in average sequence lengths of 801±219 bases and allowed gaining insight into the biodiversity as far as down to the species level (Table 1). Thereby, neonatal *Bifidobacterium* strains shared the highest sequence similarity with *B. breve*, *B. dentium*, *B. longum* and *B. pseudocatenulatum* strains deposited in GenBank. However, in contrast to maternal isolates, *B. adolescentis* or *B. catenulatum* strains were not isolated. Furthermore, common to all neonates' microbiota during the first week were high population levels of members of the Firmicutes phylum, reaching 9.99±0.36 log 16S rRNA gene copies/g feces using qPCR, but its composition differed from maternal feces. *Staphylococcus* predominated (9.37±1.60 log *tuf* copies/g) in neonatal feces, followed by *Streptococcus* (8.99±0.56 log *tuf* copies/g), while *Lactobacillus* did not seem to form stable populations and was detected only at low average levels (6.63±3.18 log 16S rRNA gene copies/g). In contrast, in maternal feces *Roseburia* spp./*Eubacterium rectale* and *Faecalibacterium prausnitzii* accounted for high Firmicutes levels, while these genera were below the detection limit of qPCR in neonatal feces. Nevertheless, anaerobic strains of the Firmicutes, i.e. *Clostridium perfringens*, *Finegoldia magna* and *Veillonella* spp. were isolated by culture at this early stage, i.e. days 4–6 of life (Table 1).

**Table 1:** Fecal strain diversity (*n* = 197), isolated from seven neonates' feces collected at 4–6 d, 9–14 d and 25–30 d postnatal, based on 16S rRNA gene sequence similarities to those deposited in the GenBank database.

Phylum	Genus	Species	Time point^1^		
			4–6 d	9–14 d	25–30 d
Actinobacteria			11	15	10
	*Bifidobacterium*	spp.	10	13	10
		*breve*	5	2	2
		*dentium*	3	3	
		*longum*		1	
		*pseudocatenulatum*		3	4
	*Eggerthella*	*lenta*		1	
	*Propionibacterium*	spp.	1	1	
		*avidum*	1		
Bacteroidetes			4	6	12
	*Bacteroides*	spp.	4	6	12
		*caccae*			1
		*dorei*			2
		*fragilis*	2	2	1
		*stercoris*	2	2	2
		*thetaiotaomicron*		2	4
Firmicutes			41	34	32
	*Bacillus*	*circulans*	1		
	*Clostridium*	spp.	4	2	2
		*butyricum*	1		
		*perfringens*	3	2	1
	*Finegoldia*	*magna*	2	1	
	*Enterococcus*	spp.	4	3	5
		*faecalis*	1		2
	*Lactobacillus*	spp.	9	5	6
		*brevis*			1
		*rhamnosus*	3	2	1
	*Lactococcus*	*lactis*	1		
	*Staphylococcus*	spp.	19	23	19
		*epidermidis*	9	5	8
		*hominis*		2	
	*Veillonella*	spp.	2		
Proteobacteria			12	11	9
	*Escherichia/Shigella*	spp.	10	10	8
	*Klebsiella*	*pneumoniae*	1	1	1

1 Number of isolates per time point.

**Figure 2:**
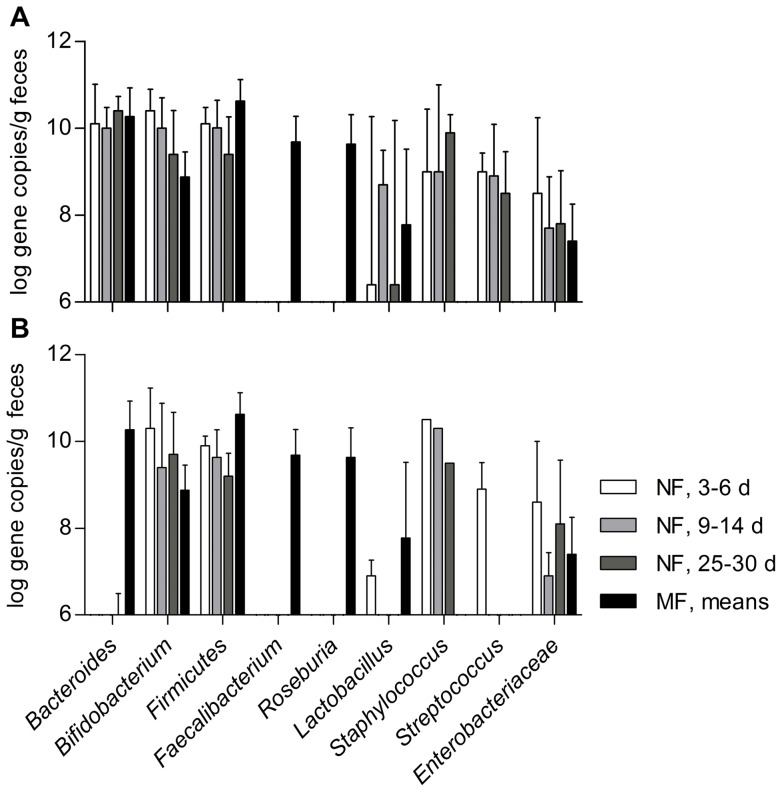
Gene copy numbers of the major gut-associated bacterial populations detected in neonatal feces (NF) using qPCR. Values are expressed as means ± SD at each of the three sampling points, i.e. days 4–6, 9–14 and 25–30 postnatal in neonates harboring high (**A**, *n* = 4) and low (**B**, *n* = 3) *Bacteroides* population levels, respectively; and comparison to means obtained from maternal feces (MF) (*n = *7) over the perinatal period.

Although, average population levels of *Bacteroides* tended to increase over the neonatal period (6.60±4.83, 7.77±4.06 and 8.95±2.31 log 16S rRNA gene copies/g within the first, second and fourth week postnatal, respectively) and seemed to follow a classical successional pattern, distributions across neonates were largely different and the neonates could be classified into two groups (Figure 2A and 2B). On the one hand 3 of 7 neonates showed detectable levels of *Bacteroides* only towards the fourth week of life (Figure 2B) and on the other hand, in 4 of 7 neonates maternal levels were observed within the first week of life, without significant variations over the neonatal period (Figure 2A). Strain typing revealed the presence of mainly *B. fragilis* and *B. stercoris*, and furthermore that those species were isolated from neonatal and corresponding maternal feces (Table 1).

### Pyrosequencing

**Figure 3:**
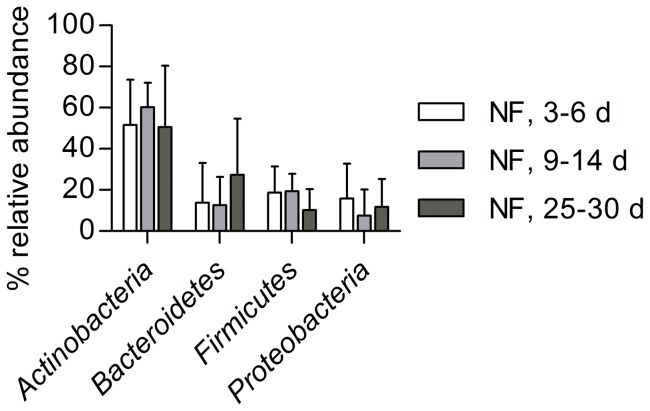
Relative 16S rRNA gene abundances of the four major phyla detected in neonatal feces (NF) using pyrosequencing. Values are expressed as means ± SD at each of the three sampling points, i.e. days 4–6, 9–14 and 25–30 postnatal.

**Figure 4:**
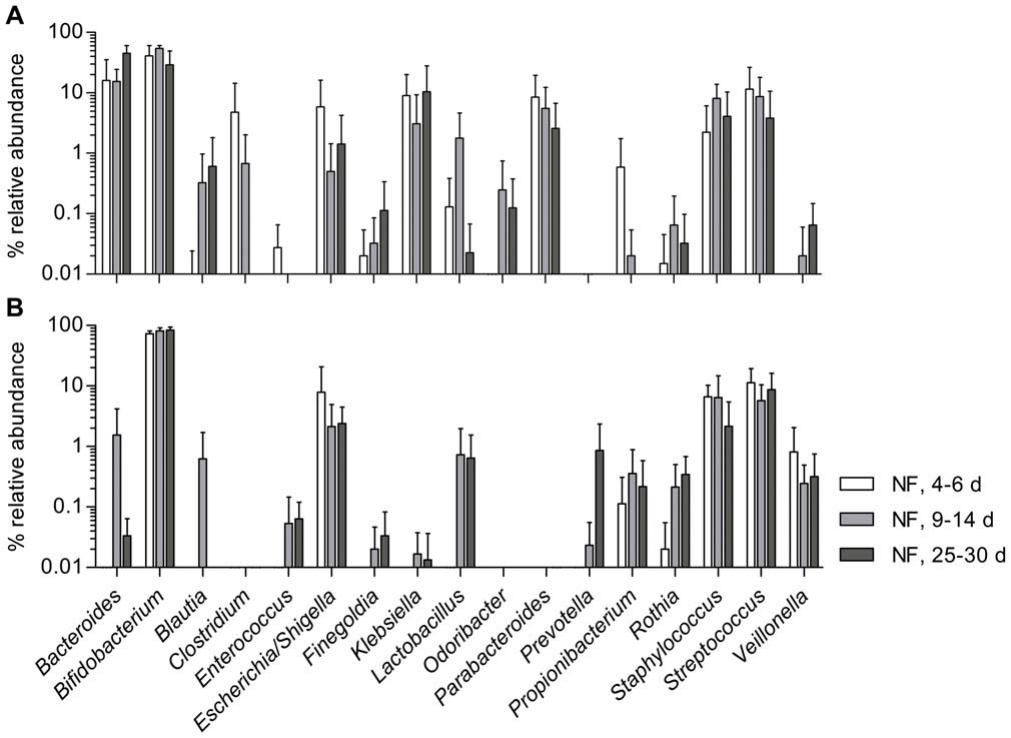
Relative 16S rRNA gene abundances of the 17 most abundant genera detected in neonatal feces (NF) using pyrosequencing. Values are expressed as means ± SD at each of the three sampling points, i.e. days 4–6, 9–14 and 25–30 postnatal in neonates harboring high (**A**, *n* = 4) and low (**B**, *n* = 3) *Bacteroides* population levels, respectively.

Pyrosequencing analysis performed on neonatal fecal DNA generated an average of 10850±2275 high-quality, taxonomically classifiable 16S rRNA gene sequences with mean read lengths of 254.7±0.9 nt (range 240–367 nt). Richness and diversity remained relatively stable over the neonatal period and no significant variations could be calculated (Chao1: 401±110, 482±159 and 440±103; Shannon: 4.02±0.26, 4.06±0.32 and 4.08±0.32, each at 0.03 OTU cutoff, within the first, second and fourth week postnatal, respectively) (Table S2). Mean read abundances at each of the three successional fecal sampling points were in general agreement with the population pattern assessed by culture and qPCR, and allowed gaining a broader view of the neonatal microbial diversity; represented on the one hand at the phylum-level (Figure 3) and on the other hand at the genus-level (Figure 4A and 4B). Over the neonatal period the Actinobacteria phylum was significantly higher than all other phyla, ranging from 49–60%, while among the Bacteroidetes, Firmicutes and Proteobacteria variations in abundance were not significant (Figure 3). Sequence assignments on lower taxonomic levels revealed that the phylum Actinobacteria was largely made up of the family Bifidobacteriaceae and consisting mainly of the genus *Bifidobacterium*. The Bacteroidetes phylum comprised mainly members of the families Bacteroidaceae and Porphyromanaceae and more specifically the genera *Bacteroides*, *Parabacteroides*, and lower abundances of *Odoribacter* and *Prevotella* (Figure 4A). As detected by both culture and qPCR, *Streptococcus* and *Staphylococcus* reached the highest relative abundances within the Firmicutes phylum, while *Lactobacillus* was detected at lower levels with large fluctuations. Furthermore, anaerobic members of this phylum, *Clostridium*, *Veillonella* and *Finegoldia* spp. were identified by isolation and 16S rRNA gene sequencing (Table 1), and by pyrosequencing at relatively low abundances (Figure 4A and 4B), while *Roseburia*, *Eubacterium*, *Faecalibacterium*, and *Ruminococcus* populations were not detected during the neonatal period by any method used. Proteobacteria consisted mainly of members of the Enterobacteriaceae, including *Escherichia* and *Klebsiella* (Figure 4A). Regarding the abundance of *Bacteroides*, the grouping into low and high population levels for 3 of 7 and 4 of 7 neonates, respectively, as described with qPCR data could be confirmed (Figure 4A and 4B, respectively). A high abundance of *Bacteroides* seemed to be related to the presence of other members of the Bacteroidetes, i.e. *Parabacteroides* and *Odoribacter*, and also to the opportunistic pathogenic genus *Klebsiella*, although the correlation was not significant (*P* = 0.073) (Figure 4). High *Bacteroides* levels also correlated with low *Bifidobacterium* levels and vice versa (Spearman *r = *−0.8281, *P<*0.0001, *n = *23), which was most apparent when analyzing first-week samples of two infants that could not be further followed up due to stringent inclusion criteria: feces of the first one harbored very low levels of *Bifidobacterium* (0.01%) and almost 90% of *Bacteroides* at postnatal day 3, while the opposite was observed for the second neonate with 88% and 0.07%, respectively.

## Discussion

The present study investigated the establishment of pioneer gut microbiota, which is increasingly recognized as a crucial stage in neonatal development influencing gut mucosal structure and immunity and thereby health and disease throughout life. We focused on healthy vaginally-delivered, exclusively breast-fed neonates, assuming their successional population pattern to be the most desirable in regard to later health status due to low incidence of morbidity and mortality, and immune-related disorders compared to other modes of delivery and feeding. Therefore, neonatal feces sampled from seven neonates at days 4–6, 9–14 and 25–30 postnatal were analyzed using a comprehensive analysis approach complementing anaerobic culture with state-of-the-art molecular methods in order to provide stronger evidence on microbiota composition, due to the fact that each method has its own limitations.

Quantitative data obtained from both culture and qPCR for total anaerobes and total bacteria, respectively, demonstrated that microbiota establishment occurred rapidly to maternal levels in all neonates' feces and remained relatively stable throughout the neonatal period, showing that maximum bacterial densities are reached as early as day four of life. Interestingly, total anaerobes already outnumbered total facultative anaerobes by factor >60, which was not expected to occur at this early stage, as ratios of 0.1 and 4 during the first week and fourth week of life, respectively, have been reported previously [21]. These differences may be explained by different culture methods and our efforts in maintaining anaerobic conditions throughout sampling, transport and culture. However, since no samples were collected before day four of life, a gradual increase in bacterial densities, as reported by Palmer using qPCR [10], and thus an initial dominance by facultative anaerobes cannot be excluded; but it appears that such populations are not able to dominate the microbiota by the time a maximum density is reached.

Despite individual-specific successional population pattern, the predominance of anaerobes from as early as day four of life was largely due to high levels of *Bifidobacterium*, acting as pioneer bacteria. Unexpectedly, early onset of *Bacteroides* establishment and stability over the neonatal period was detected in 4 of 7 neonates. In contrast, in 3 of 7 neonates this genus seemed to follow a classical successional population pattern with subdominant levels appearing late during the neonatal period. While early establishment of *Bifidobacterium* has been reported in previous studies assessing the microbiota of breast-fed neonates within the first week of life [8], [17], [41]–[43], published data on the onset of other anaerobes such as *Bacteroides* and their population levels are ambiguous. For instance, using culture, Adlerberth [7] reported that the *Bacteroides* population would establish much later than the *Bifidobacterium* population. Palmer [10], when using 16S rRNA gene hybridization microarrays, stated that the timing of establishment of this genus was largely individual-specific and that consistent population levels were detected in nearly all of their study participants only by the age of one year. Interestingly, neonates harboring high levels of *Bifidobacterium* harbored lower levels of *Bacteroides* and vice versa. Besides environmental and genetic host factors, the inverse correlation between these two major anaerobic gut populations may result from differences in the composition of the maternal microbiota, especially the initial inoculum transferred by contact with the vaginal (and anal) microbiota during delivery, as well as the bacterial inoculum provided continuously by breast milk. Furthermore, differences in the nutritional composition of breast milk may impact the neonatal microbiota, such as the wide range of human milk oligosaccharides (HMO) and lipids, and thus the competition for these substrates. In this regard, inter-individual differences in composition of human milk oligosaccharides, such as the ratio of fucosylated to sialyated oligosaccharides may be an important selective factor, as it has been shown that the ability to grow on HMO is strain-dependent [44]–[46]. However, despite similar metabolic functions, gram-positive bacteria elicit different immune responses than gram-negatives [8]. Therefore a change in the *Bifidobacterium* to *Bacteroides* ratio may result in different susceptibilities to inflammation and affect later health. In this regard conflicting results have been published previously: early establishment of a *Bacteroides* population has been associated with possible asthma in later life [47], while other studies suggested positive, protective effects on mucosal immunity [48], [49].

The *Bifidobacterium* species identified most frequently by strain typing was *B. breve*, which has been reported typical for the microbiota of breast-fed infants [7]. However, no typical maternal species, such as *B. adolescentis*, were isolated in the present study, suggesting that the early *Bifidobacterium* population is transient. In contrast, the *Bacteroides* species isolated within the neonatal period, including *B. fragilis*, *B. stercoris*, *B. thetaiotaomicron*, were also detected in corresponding maternal feces, suggesting that these pioneer bacteria remain part of the adult microbiota.

Also common to all neonates’ fecal microbiota was that facultative anaerobic populations were largely made up of *Staphylococcus* and *Streptococcus*, resulting in similar levels of Firmicutes as detected in maternal feces. However, in maternal feces this phylum comprised the major butyrate-producing members of the clostridial cluster IV and XIV, i.e. *Faecalibacterium* and *Roseburia* spp., respectively, which are apparently not able to reach detectable population levels in breast-fed neonates during the neonatal period. The timing by which these butyrate-producers increased in population density should be further studied, since besides their multiple functions they are essential for a healthy physiology of colonocytes [50].

Ultimately, pyrosequencing of neonatal fecal DNA was carried out to qualitatively confirm the data obtained by culture and qPCR, and to gain a broader view on bacterial diversity, due to the fact that on the one hand fastidious anaerobes may have escaped cultivation, and on the other hand, that detection of diversity comparable to that obtained by pyrosequencing goes beyond the scope of qPCR. Considering the evolution of the diversity indices based on OTU identified using pyrosequencing, only a slight tendency towards an increased diversity was observed in all but one neonate during the neonatal stage. This indicates that an initial diversity is reached rather rapidly and that further diversification of the pioneer microbiota is a slow process, at least until weaning. De Filippo [28] reported a median abundance of *Bacteroides* of approximately 30% in a cohort of European children (1–6 years of age), which is only slightly higher than the abundances reached in our study cohort already in week four postnatal.

Results obtained from pyrosequencing were qualitatively in agreement with those obtained from culture and qPCR methods, i.e. *Bifidobacterium* and *Bacteroides* were the major pioneer populations, followed by members of the Firmicutes, *Staphylococcus* and *Streptococcus*, and lower numbers of Enterobacteriaceae. However, quantitative comparisons should be taken with caution, since each of the methods used has its advantages, as well as its inherent biases (e.g. highest sensitivity in culture, but medium selectivity bias; highest quantitative accuracy in qPCR, and broadest taxon quantitation by pyrosequencing, but relatively low sensitivity; multiple 16S rRNA gene copy bias and primer specificity bias in molecular methods). Thus, the main point for using multiple methods is complementarity, although quantitative similarities were observed for the dominant populations, such as *Bifidobacterium* and *Bacteroides*, which were cultured and isolated, as well as detected by qPCR and pyrosequencing at high levels. However, for subdominant populations concordance becomes less clear due to the different sensitivities of the methods. For instance, the low and individual-specific levels of *Lactobacillus* observed using qPCR confirm previous research suggesting that this genus is unable to form stable population during early life [8]. It should be noted however that the culture medium used was highly selective, resulting in almost all isolates being *Lactobacillus* spp., but on the downside some strains may have escaped culture. Similarly, the low *Lactobacillus* population levels detected by qPCR in some neonates fell below the detection limit of pyrosequencing.

Furthermore, pyrosequencing revealed that in the group of neonates showing high levels of *Bacteroides*, also other anaerobes of the Bacteroidetes phylum were detected as pioneer bacteria, such as *Parabacteroides*, *Prevotella* and *Odoribacter*. At the same time higher relative abundances of opportunistic pathogens, notably *Klebsiella* and *Clostridium* were detected within this group compared to the group showing low and late *Bacteroides* levels. It remains, however, unclear whether these differences in population pattern are desirable for the development of immunity and if the higher levels of *Bifidobacterium* in the latter group were responsible for the absence of such opportunistic pathogens.

In conclusion, this study contributes further to the understanding of early neonatal gut microbiota establishment and points out that anaerobes may become dominant early in the successional process. While our data are in agreement with previous studies showing that *Bifidobacterium* are able to reach high levels in vaginally-delivered, breast-fed neonates, the presence of Bacteroidetes as pioneer bacteria in the majority of neonates studied demonstrates that adult-type strict anaerobes may reach adult-like population densities already within the first week of life. Consequently the switch from facultative to strict anaerobes may occur earlier than previously assumed. As only seven neonates were included in the present study, however, the observed differences in gut colonization pattern should be verified in a larger cohort study. Furthermore, the major adult-type, butyrate-producing anaerobic populations, *Roseburia* and *Faecalibacterium*, which are essential for colonic health, remained undetectable during the neonatal period by any of the methods used. These populations have never been extensively studied in neonates and the fact that other strict anaerobes are able to establish early suggest that their establishment is rather limited to the absence of metabolic cross-feeding between members of the microbiota than to the absence of anaerobic conditions. The impact of the nutritional components provided by breast milk (e.g. HMO) on the timing of establishment of anaerobic pioneer bacteria, as well as opportunistic pathogens (e.g. *Klebsiella*) should be further studied in regard to priming of the gut-associated immune system and consequences on later health outcome; and eventually open the field for nutritional modulation if necessary.

## Supporting Information

Figure S1 **Bacterial populations detected in feces collected from seven neonates (A–G) at days 4–6, 9–14 and 25–30 postnatal (NF1, NF2 and NF3, respectively), using culture, qPCR and pyrosequencing (I, II and III, respectively).** Values are means of duplicates and triplicates for culture and qPCR, respectively, and single values for pyrosequencing. Neonates A–D and E–G fall under the groups with high and low *Bacteroides* population levels, respectively. A comparison to corresponding mean maternal fecal population levels (MF) is given for culture and qPCR. In panel E II, values for NF1 and NF2 have been omitted due to the presence of PCR inhibitors.
(DOC)

Table S1 **Oligonucleotide primers used for quantitative PCR.**
(DOC)

Table S2 **16S rRNA gene read numbers, percentage of reads taxonomically classified, and richness (Chao1) and diversity (Shannon) indexes at OTU distance cutoffs of 0.03, 0.05 and 0.10, obtained by 454–pyrosequencing of neonatal fecal DNA.**
(DOC)
