# Supplementary material for: New Insights in Gut Microbiota Establishment in Healthy Breast Fed Neonates
Source: PLoS One. 2012 Aug 30;7(8):e44595. doi: 10.1371/journal.pone.0044595 (PMC3431319; doi:10.1371/journal.pone.0044595)
Supplement: Figure S1 — Bacterial populations detected in feces collected from seven neonates (A–G) at days 4–6, 9–14 and 25–30 postnatal (NF1, NF2 and NF3, respectively), using culture, qPCR and pyrosequencing (I, II and III, respectively). Values are means of duplicates and triplicates for culture and qPCR, respectively, and single values for pyrosequencing. Neonates A–D and E–G fall under the groups with high and low Bacteroides population levels, respectively. A comparison to corresponding mean maternal fecal population levels (MF) is given for culture and qPCR. In panel E II, values for NF1 and NF2 have been omitted due to the presence of PCR inhibitors. (DOC) [file pone.0044595.s001.doc]

**Figure S1. Bacterial populations detected in feces collected from seven neonates (A-G) at days 4-6, 9-14 and 25-30 postnatal (NF1, NF2 and NF3, respectively), using culture, qPCR and pyrosequencing (I, II and III, respectively).** Values are means of duplicates and triplicates for culture and qPCR, respectively, and single values for pyrosequencing. Neonates A-D and E-G fall under the groups with high and low *Bacteroides* population levels, respectively. A comparison to corresponding mean maternal fecal population levels (MF) is given for culture and qPCR. In panel E II, values for NF1 and NF2 have been omitted due to the presence of PCR inhibitors.
