# Supplementary material for: New Insights in Gut Microbiota Establishment in Healthy Breast Fed Neonates
Source: PLoS One. 2012 Aug 30;7(8):e44595. doi: 10.1371/journal.pone.0044595 (PMC3431319; doi:10.1371/journal.pone.0044595)
Supplement: Table S1 — Oligonucleotide primers used for quantitative PCR. (DOC) [file pone.0044595.s002.doc]

**Table S1**. Oligonucleotide primers used for quantitative PCR.

| Target group | Primer and sequence (5’-3’) | Target gene and standard | Reference |
| --- | --- | --- | --- |
| Total bacteria | Eub338F, ACTCCTACGGGAGGCAGCAG  Eub518R, ATTACCGCGGCTGCTGG | 16S rRNA, Plasmid pLME21 containing *Bifidobacterium lactis* DSM10140T 16S rRNA gene |  |
| *Bifidobacterium* spp. | xfp-fw, ATCTTCGGACCBGAYGAGAC  xfp-rv, CGATVACGTGVACGAAGGAC | *xfp*^1^, *Bifidobacterium longum* DSM20219T *xfp* amplicon |  |
| *Bacteroides* spp. | Bac303F, GAAGGTCCCCCACATTG  Bfr-Fermrev, CGCKACTTGGCTGGTTCAG | 16S rRNA, *Bacteroides thetaiotaomicron* DSM2079T 16S rRNA gene |  |
| Firmicutes | Firm934F, GGAGYATGTGGTTTAATTCGAAGCA  Firm1060R, AGCTGACGACAACCATGCAC | 16S rRNA, *Roseburia intestinalis* DSM14610T 16S rRNA gene |  |
| *Lactobacillus*/ *Leuconostoc*/ *Pediococccus* spp. | F_Lacto 05, AGCAGTAGGGAATCTTCCA  R_Lacto 04, CGCCACTGGTGTTCYTCCATATA | 16S rRNA, *Lactobacillus delbrueckii* DSM20081T 16S rRNA gene |  |
| *Roseburia* spp./ *Eubacterium rectale* | RrecF, GCGGTRCGGCAAGTCTGA  Rrec630mR, CCTCCGACACTCTAGTMCGAC | 16S rRNA, *Roseburia intestinalis* DSM14610T 16S rRNA gene |  |
| *Faecalibacterium prausnitzii* | Fprau223F, GATGGCCTCGCGTCCGATTAG  Fprau420R, CCGAAGACCTTCTTCCTCC | 16S rRNA, *Faecalibacterium prausnitzii* DSM17677 16S rRNA gene |  |
| *Streptococcus* spp. | Tuf-Strep-1, GAAGAATTGCTTGAATTGGTTGAA  Tuf-Strep-R, GGACGGTAGTTGTTGAAGAATGG | *tuf*^2^, *Streptococcus mitis* DSM12643T *tuf* amplicon |  |
| *Staphylococcus* spp. | TStaG422, GGCCGTGTTGAACGTGGTCAAATCA  TStag765, TYACCATTTCAGTACCTCTGGTAA | *tuf*, *Staphylococcus epidermidis* DSM20044T *tuf* amplicon |  |
| Enterobacteriaceae | Eco1457F, CATTGACGTTACCCGCAGAAGAAGC  Eco1652R, CTCTACGAGACTCAAGCTTGC | 16S rRNA, *Escherichia coli* DSM5698 16S rRNA gene |  |

^1^ xylose-5-phosphate/ fructose-6-phosphate phosphoketolase gene

^2^ elongation factor Tu gene

1. Lane DJ (1991) 16S/23S rRNA sequencing. In: Stackebrandt E, Goodfellow M, editors. Nucleic acid techniques in bacterial systematics. Chichester, England: John Wiley & Sons. pp. 115-175.

2. Muyzer G, de Waal EC, Uitterlinden AG (1993) Profiling of complex microbial populations by denaturing gradient gel electrophoresis analysis of polymerase chain reaction-amplified genes coding for 16S rRNA. Appl Environ Microbiol 59: 695-700.

3. Cleusix V, Lacroix C, Dasen G, Leo M, Le Blay G Comparative study of a new quantitative real-time PCR targeting the xylulose-5-phosphate/fructose-6-phosphate phosphoketolase bifidobacterial gene (xfp) in faecal samples with two fluorescence in situ hybridization methods. J Appl Microbiol 108: 181-193.

4. Manz W, Amann R, Ludwig W, Vancanneyt M, Schleifer KH (1996) Application of a suite of 16S rRNA-specific oligonucleotide probes designed to investigate bacteria of the phylum cytophaga-flavobacter-bacteroides in the natural environment. Microbiology 142 ( Pt 5): 1097-1106.

5. Ramirez-Farias C, Slezak K, Fuller Z, Duncan A, Holtrop G, et al. (2009) Effect of inulin on the human gut microbiota: stimulation of Bifidobacterium adolescentis and Faecalibacterium prausnitzii. Br J Nutr 101: 541-550.

6. Guo X, Xia X, Tang R, Zhou J, Zhao H, et al. (2008) Development of a real-time PCR method for Firmicutes and Bacteroidetes in faeces and its application to quantify intestinal population of obese and lean pigs. Lett Appl Microbiol 47: 367-373.

7. Furet JP, Firmesse O, Gourmelon M, Bridonneau C, Tap J, et al. (2009) Comparative assessment of human and farm animal faecal microbiota using real-time quantitative PCR. FEMS Microbiol Ecol 68: 351-362.

8. Walker AW, Duncan SH, McWilliam Leitch EC, Child MW, Flint HJ (2005) pH and peptide supply can radically alter bacterial populations and short-chain fatty acid ratios within microbial communities from the human colon. Appl Environ Microbiol 71: 3692-3700.

9. Bartosch S, Fite A, Macfarlane GT, McMurdo ME (2004) Characterization of bacterial communities in feces from healthy elderly volunteers and hospitalized elderly patients by using real-time PCR and effects of antibiotic treatment on the fecal microbiota. Appl Environ Microbiol 70: 3575-3581.

10. Wang RF, Cao WW, Cerniglia CE (1996) PCR detection and quantitation of predominant anaerobic bacteria in human and animal fecal samples. Appl Environ Microbiol 62: 1242-1247.

11. Collado MC, Delgado S, Maldonado A, Rodriguez JM (2009) Assessment of the bacterial diversity of breast milk of healthy women by quantitative real-time PCR. Lett Appl Microbiol 48: 523-528.

12. Martineau F, Picard FJ, Ke D, Paradis S, Roy PH, et al. (2001) Development of a PCR assay for identification of staphylococci at genus and species levels. Journal of Clinical Microbiology 39: 2541-2547.
