# Supplementary material for: New Insights in Gut Microbiota Establishment in Healthy Breast Fed Neonates
Source: PLoS One. 2012 Aug 30;7(8):e44595. doi: 10.1371/journal.pone.0044595 (PMC3431319; doi:10.1371/journal.pone.0044595)
Supplement: Table S2 — 16S rRNA gene read numbers, percentage of reads taxonomically classified, and richness (Chao1) and diversity (Shannon) indexes at OTU distance cutoffs of 0.03, 0.05 and 0.10, obtained by 454–pyrosequencing of neonatal fecal DNA. (DOC) [file pone.0044595.s003.doc]

**Table S2.** 16S rRNA gene read numbers, percentage of reads taxonomically classified, and richness (Chao1) and diversity (Shannon) indexes at OTU distance cutoffs of 0.03, 0.05 and 0.10, obtained by 454-pyrosequencing of neonatal fecal DNA.

| Sample^1^ | Read number^2^ | Taxonomic classification^3^ (%) | | | OTU | | | Chao index | | | Shannon index | | |
| --- | --- | --- | --- | --- | --- | --- | --- | --- | --- | --- | --- | --- | --- |
|  |  | Phylum | Family | Genus | 0.03 | 0.05 | 0.1 | 0.03 | 0.05 | 0.1 | 0.03 | 0.05 | 0.1 |
| A1 | 13320 | 97.6 | 94.9 | 92.4 | 284 | 119 | 45 | 564 | 233 | 56 | 2.59 | 2.03 | 1.84 |
| A2 | 11258 | 98.5 | 95.8 | 94.3 | 239 | 87 | 29 | 568 | 128 | 33 | 2.13 | 1.56 | 1.35 |
| A3 | 7684 | 98.8 | 94.7 | 92.1 | 230 | 84 | 33 | 453 | 169 | 45 | 2.66 | 1.92 | 1.64 |
| B1 | 11738 | 99.6 | 98.2 | 97.9 | 240 | 93 | 34 | 583 | 199 | 39 | 2.53 | 2.10 | 1.34 |
| B2 | 15016 | 99.2 | 96.9 | 96.0 | 369 | 165 | 72 | 908 | 319 | 144 | 2.73 | 2.39 | 1.73 |
| B3 | 13884 | 99.3 | 96.6 | 95.0 | 324 | 150 | 69 | 549 | 228 | 177 | 2.69 | 2.34 | 1.84 |
| C1 | 11439 | 98.1 | 97.7 | 94.9 | 179 | 67 | 20 | 369 | 125 | 35 | 2.13 | 1.39 | 1.21 |
| C2 | 10488 | 98.8 | 97.3 | 95.8 | 196 | 86 | 43 | 373 | 119 | 58 | 2.55 | 2.00 | 1.82 |
| C3 | 13761 | 99.6 | 97.9 | 97.2 | 124 | 48 | 30 | 248 | 71 | 37 | 1.59 | 1.20 | 1.14 |
| D1 | 6186 | 99.3 | 98.8 | 94.4 | 115 | 40 | 23 | 258 | 53 | 24 | 1.96 | 1.55 | 1.44 |
| D2 | 8752 | 99.4 | 97.8 | 96.3 | 164 | 67 | 35 | 305 | 154 | 44 | 1.92 | 1.43 | 1.33 |
| D3 | 8624 | 99.4 | 99.0 | 97.4 | 148 | 68 | 35 | 421 | 151 | 41 | 1.80 | 1.46 | 1.39 |
| E1 | 10793 | 99.2 | 97.4 | 96.1 | 168 | 58 | 26 | 420 | 100 | 32 | 1.90 | 1.29 | 0.98 |
| E2 | 10406 | 98.1 | 97.1 | 66.0 | 171 | 66 | 24 | 446 | 98 | 31 | 2.26 | 1.65 | 1.28 |
| E3 | 11872 | 98.3 | 97.5 | 74.6 | 201 | 84 | 37 | 382 | 117 | 43 | 2.08 | 1.58 | 1.24 |
| F1 | 12791 | 99.1 | 98.3 | 96.9 | 180 | 54 | 16 | 390 | 97 | 16 | 1.76 | 1.25 | 0.72 |
| F2 | 12562 | 99.0 | 96.4 | 95.0 | 341 | 166 | 78 | 719 | 263 | 128 | 2.72 | 2.01 | 1.17 |
| F3 | 9338 | 98.7 | 97.6 | 95.8 | 247 | 131 | 61 | 509 | 239 | 100 | 2.48 | 1.85 | 1.06 |
| G1 | 7060 | 98.6 | 96.6 | 68.4 | 181 | 67 | 26 | 391 | 112 | 41 | 2.02 | 1.60 | 1.24 |
| G2 | 10578 | 99.8 | 82.8 | 82.1 | 175 | 69 | 26 | 322 | 120 | 28 | 2.15 | 1.03 | 0.86 |
| G3 | 10308 | 99.7 | 97.0 | 95.3 | 203 | 71 | 29 | 397 | 109 | 56 | 2.24 | 1.40 | 0.44 |

^1^ A-G stand for the seven neonates, 1-3 for the different sampling points, i.e. 4-6 d, 9-14 d and 25-30 d postnatal, respectively

^2^ number of reads that passed quality control

^3^ classified using Ribosomal Database Project (RDP) classifier at a confidence threshold of 80%
